# Characteristics and influencing factors of anticipated HIV stigma among HIV‐negative/unknown MSM in China: A regression mixture model

**DOI:** 10.1002/brb3.3472

**Published:** 2024-03-29

**Authors:** Zhenwei Dai, Yijin Wu, Xin Liu, Jiaqi Fu, Mingyu Si, Xu Chen, Hao Wang, Weijun Xiao, Yiman Huang, Fei Yu, Guodong Mi, Xiaoyou Su

**Affiliations:** ^1^ School of Population Medicine and Public Health Chinese Academy of Medical Sciences & Peking Union Medical College Beijing China; ^2^ Danlan Public Welfare Beijing China

**Keywords:** anticipated stigma, china, HIV, HIV‐negative/unknown MSM, regression mixture model

## Abstract

**Background:**

Anticipated HIV stigma among men who have sex with men's (MSM) has a severe negative effect on their physical and mental health wellbeing and hence requires specific attention. The current study aims to identify the characteristics and the psychosocial influencing factors of anticipated HIV stigma in MSM using regression mixture model (RMM) and to determine the cut‐off point of the seven‐item Anticipated HIV Stigma Questionnaire (AHSQ) using the receiver operating characteristic (ROC) analysis.

**Methods:**

A cross‐sectional study was conducted among HIV‐negative/unknown MSM from Blued online platform in China from December 16th, 2020 to March 1st, 2021, enrolling 1394 participants. Data were collected on demographic characteristics, perceived social support, anticipated HIV stigma, depressive symptoms, and HIV knowledge. Latent profile analysis was performed to identify different profiles of anticipated HIV stigma level. Chi‐square test, analysis of variance, and RMM analysis were conducted to explore the influencing factors in different profiles. ROC analyses were carried out to identify the cut‐off value of anticipated stigma.

**Results:**

Among the participants, three profiles of anticipated stigma were identified: “low anticipated HIV stigma” (12.0%), “moderate anticipated HIV stigma” (52.1%), and “severe anticipated HIV stigma” (35.9%). RMM analysis showed that higher income and higher levels of knowledge were positively associated with moderate anticipated HIV stigma, whereas full‐time job and social support were negatively associated with moderate anticipated HIV stigma; higher income, depressive symptoms, and knowledge were positively associated with severe anticipated HIV stigma, whereas minor ethnicity and social support were negatively associated with severe anticipated HIV stigma. ROC curve of the AHSQ showed that the optimal cut‐off value of ≥16 could indicate positive anticipated HIV stigma.

**Conclusion:**

The study focuses on the level of anticipated HIV stigma and its psycho‐socio influencing factors among HIV‐negative/unknown MSM. It provides evidence for implementing relevant psychological interventions to HIV‐negative/unknown MSM.

## Graphical abstract

Profiles of anticipated HIV stigma in MSM.

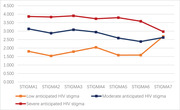

## 1 INTRODUCTION

The AIDS epidemic is a public health threat that the global community is jointly facing (De Lay et al., 2021; Lancet, 2017). According to the report by UNAIDS (Joint United Nations Program on HIV/AIDS), there were nearly 38.4 million people living with HIV (PLWH) and 1.5 million new HIV infections in 2021 worldwide (UNAIDS, 2022a). In China, 60154 people were newly infected with HIV, with an incidence rate of 4.27 in 100,000 people in 2021 (Statistics, 2022). In most countries with sizable and noticeable men who have sex with men (MSM) communities, an increasing proportion of MSM have been diagnosed with HIV since the late 1990s (Gao et al., 2009; Marcus et al., 2006). In China, MSM also have disproportionately high burdens of HIV infections compared with other populations due to risky sexual behaviors (Beyrer et al., 2016; Kaufman & Jing, 2002; Van Der Zee et al., 2023; Zerdali et al., 2023; Zhang & Chu, 2005; Zhang et al., 2008). According to the most recent Global AIDS Monitoring in 2022, HIV prevalence among MSM in China was 5.4% (WHO, 2022).

Evidence has consistently demonstrated the effectiveness of various measures and strategies in preventing HIV infection. Notably, the utilization and promotion of pre‐exposure prophylaxis (PrEP) and postexposure prophylaxis (PEP), as well as the introduction and dissemination of the concept of “treatment as prevention,” have significantly promoted global efforts in HIV control and prevention (Elliott et al., 2019). However, stigma has been identified as one of the primary obstacles in these efforts. Research indicates that stigma was not only associated with lower use of PrEP and PEP, causing the further transmission of HIV, but also adversely affects the adherence to antiretroviral therapy, leading to viral suppression failure in PLWH (Brooks et al., 2019; Camacho et al., 2020; Koblin et al., 2018). This may cause antiviral resistance, which seriously affects the health of PLWH. Therefore, addressing issues of stigma is of critical importance at this current stage.

MSM face severe psychological and social problems. MSM are disproportionately affected by various psychological problems, including depression, anxiety, distress, trauma, obsessive‐compulsive behavior, interpersonal sensitivity, and substance use (Batchelder et al., 2017; Liu et al., 2018). For MSM, stigma and discrimination is an important social process related to power, domination, and inequality (Parker & Aggleton, 2003). The HIV stigma framework defines stigma in three mechanisms: enacted stigma, involving the discrimination they experience in the community; anticipated stigma, involving the prediction of being discriminated and prejudiced; and internalized stigma, involving the endorsed negative beliefs and labels the stigmatized person applied to oneself (Earnshaw & Chaudoir, 2009). HIV‐positive MSM who have stigma experience tend to have higher prevalence of HIV‐transmission risk behaviors and poorer self‐reported health status (Bogart et al., 2013; Hatzenbuehler et al., 2011; Lyons et al., 2023; Tao et al., 2022). Additionally, stigma‐related experiences might increase MSM's possibility of mental health problems, like depressive symptoms, anxiety, and low self‐esteem (Mitzel et al., 2015; Murphy et al., 2018; Yan et al., 2019; Zhai et al., 2023).

Stigma not only harms the HIV‐positive MSM but also has serious negative impact on HIV‐negative/unknown MSM. Among the HIV‐negative/unknown MSM, anticipated stigma has been prevalent and done harm to their own health as well as the population's HIV prevention. Anticipated HIV stigma is an individual's expectation that if they become HIV infected, they would face prejudice, rejection, and bias (Liu et al., 2020). Anticipated stigma overburdens one's physical functioning and even is a potential chronic stressor (Link & Phelan, 2006; Okonkwo et al., 2022). It might also cause the HIV‐negative MSM's poorer overall physical health perceptions (Brewer et al., 2020). Additionally, anticipated stigma predicts negative mental health, like less social support or lower trust in healthcare workers (Starks et al., 2013; Turan et al., 2017). Therefore, MSM's anticipated HIV stigma has a severe negative effect on their physical and mental health status and hence requires our attention.

Stigma would be affected by various social‐psycho factors. From the psychological perspective, a study on children affected by HIV/AIDS aged 6–12 in rural China showed that perceived stigma was influenced by depressive symptoms (Chi et al., 2014). Another study on PLWH in Hong Kong, China also revealed that positive mental health status buffered the effects of self‐stigma (Yang & Mak, 2017). From the social perspective, a meta‐analysis suggested that social support could protect against HIV‐related stigma in PLWH (Armoon et al., 2022). In addition, a study on rural‐to‐urban migrants in China indicated that HIV‐related knowledge could have an influence on their stigma, further affecting their willingness to HIV serostatus disclosure (Yang et al., 2006). A meta‐analysis also demonstrated that programs to improve HIV‐related knowledge are effective to reduce HIV stigma (Mak et al., 2017). Thus, anticipated stigma might also be affected by HIV‐negative/unknown MSM's mental health status, social support, and HIV‐related knowledge and awareness.

In this study, we used a seven‐item Anticipated HIV Stigma Questionnaire (AHSQ) to assess the anticipated stigma among Chinese HIV‐negative/unknown MSM (Liu et al., 2020). Despite reliable and valid among Chinese HIV‐negative/unknown MSM, this scale has no cut‐off point currently, which makes it unintuitive to precisely and quantitatively evaluate the anticipated stigma among HIV‐negative/unknown MSM. In addition, the characteristics and prevalence of anticipated HIV stigma among MSM and its psychosocial influencing factors remain ambiguous. Furthermore, most previous studies emphasized the recursive effect of perceived stigma on mental health without considering the potential non‐recursive circle between mental health and anticipated HIV stigma among MSM. Latent profile analysis (LPA) is a statistical method that focuses on individuals to predict subpopulation profiles with the measured continuous factors (Choi et al., 2021). As a model‐based methodology, LPA could provide more accurate classification compared with simple hierarchical classification (Pastor et al., 2007). Additionally, regression mixture model (RMM) is a statistical model that could involve covariates as predictors for LPA, which could help to further determine the influencing factors of the characteristics identified by LPA (Lamont et al., 2016). RMM has been employed in the analysis of stigma in certain populations (Loch et al., 2014; Wu et al., 2023). The specific objectives of current study are to identify the characteristics of anticipated HIV stigma in MSM using LPA; to explore the psychosocial influencing factors of anticipated HIV stigma in MSM using RMM based on the result of LPA; and to determine the cut‐off point of the AHSQ using the receiver operating characteristic (ROC) analysis for further evaluation and application, which may help healthcare professions and policymakers to deal with the anticipated stigma among MSM effectively. This study might help to better understand the situation of anticipated HIV stigma among HIV‐negative/unknown MSM, which is essential for the health promotion for MSM. Despite decades of advances in prevention and treatment, stigma and discrimination toward PLWH and key populations are a persistent barrier to ending the epidemic (UNAIDS, 2022b). This study might also help identify MSM with anticipated stigma to achieve the goal of the United Nations to end HIV‐related stigma and discrimination.

## 2 MATERIALS AND METHODS

### 2.1 Study design and participants

Participants in this study were recruited from Blued online platform in China by convenience sampling from December 16th, 2020 to March 1st, 2021. Blued is a popular online MSM geosocial networking platform with over 40 million active users. Researchers sent study posters to potential participants’ message interface on Blued, and interested MSM then clicked on the link in the study posters to access an online questionnaire webpage.

An online questionnaire survey can ensure the integrity and anonymity of the results and let participants fill out the questionnaire in a natural and relaxed state, thus improving the authenticity of the results. Before filling in the questionnaires, participants had to read the informed consent on the first page and click a button that said “I am willing to participant in this study.” The inclusion criteria were as follows: (1) being biologically male; (2) having had oral/anal sex with men in the last year; (3) never having an HIV test or the test result was negative. The exclusion criteria were as follows: (1) below 18 years old; (2) self‐reporting HIV positive. Finally, 1396 HIV‐negative/unknown MSM that met the criteria above accomplished the questionnaire. A few logic questions were embedded in the questionnaire for quality control. The criteria for an invalid questionnaire were as follows: (1) “In the past 6 months, have you engaged in anal sex with a male?” was answered as “No,” but the response to the question “In the past 6 months, how many males have you engaged in anal sex with?” is greater than 0; or “In the past 6 months, have you engaged in anal sex with a male?” was answered as “Yes,” but the response to the question “In the past 6 months, how many males have you engaged in anal sex with?” is equal to or greater than 300. (2) The options exhibit a suspicious pattern, such as all items having identical choices. One thousand three hundred and ninety‐four were included in this study after checking their qualification. Ethical approval for the survey was obtained from the Ethics Committee of Danlan Beijing Media Limited on May 20, 2020 (Number: DLIRB202005‐01).

### 2.2 Measures

#### 2.2.1 Demographic characteristics

Participants were asked about their age, marital status, work status, ethnicity, education level, income, whether had an HIV test, whether had sex with a male in the last 6 months, whether have had sex with a female in the last 6 months, substance use in the last 6 months, and whether have the experience of using PrEP.

#### 2.2.2 Perceived social support

Perceived Social Support Questionnaire was employed to measure the social support level perceived by participants. The questionnaire was developed by Li et al. (2017). The questionnaire contained two items, and each item was 11‐point Likert scaled from 0 to 10 (0 = strongly disagree; 10 = strongly agree). Higher total scores indicated a higher level of perceived support. The Cronbach's α of the questionnaire in this study was 0.716.

#### 2.2.3 Anticipated HIV stigma

Participants’ anticipated HIV stigma was measured by AHSQ developed by Golub and Gamarel (2013). This questionnaire has been validated under Chinese culture by Liu et al. (2020). The questionnaire contained seven items and each item was rated on a four‐point Likert scale (1 = strongly disagree; 4 = strongly agree). Higher total values indicated greater anticipated stigma. The Cronbach's α of the questionnaire in this study was 0.837.

#### 2.2.4 Depressive symptoms

Center for Epidemiologic Studies Depression Scale (CES‐D_10_) was used to measure the depressive symptoms of participants in the current study. The scale was developed by Radloff (1977) and was introduced and adapted to the Chinese cultural context by Yu et al. (2013). The scale contained 10 items and each item was 4‐point Likert scaled (0 = never or seldom; 3 = most or all of the time). Higher total scores indicated higher level of depressive symptoms; a total score equal to or higher than 10 could indicate depressive symptoms. The Cronbach's α of the scale in this study was 0.863.

#### 2.2.5 HIV knowledge

HIV knowledge questionnaire (HIV‐KQ‐18) developed by Carey and Schroder (2002) was used to measure the knowledge on HIV and HIV prevention of participants. The questionnaire contains 18 items and each item was a question that need an answer between “Yes,” “No,” and “Not clear.” Participants received one‐point for each correct response, and responses were summed to derive the total score for each participant. Higher total scores indicated higher level of HIV knowledge. The Cronbach's α of the questionnaire in this study was 0.759.

### 2.3 Statistical analysis

Descriptive analyses were performed to describe the participants’ demographic characteristics. LPA is a person‐centered statistical method that divides a population into several subpopulations based on a set of continuous variables (Berlin et al., 2014; Bondjers et al., 2018; Lubke & Muthen, 2005). LPA has been universally used to identify the symptom characteristics as well as to calculate and determine optimal cut‐off points for assessment instruments and scales with relatively low misclassification rate and more reasonable results than other approaches (Fu et al., 2022; Li et al., 2020; Magidson & Vermunt, 2002; Meehl & Yonce, 1996; Van Smeden et al., 2014; Yu et al., 2020). Therefore, LPA was conducted to identify the characteristics of anticipated HIV stigma among MSM in this study. In LPA, robust maximum likelihood estimation was employed to estimate the parameters. The Lo‐Mendell‐Rubin (LMR) and the bootstrap likelihood ratio test (BLRT) were performed to compare the model fit improvement between models with k classes and k‐1 classes, significant *p* values indicated a better model fit with k classes. The optimal number of classes was evaluated by the entropy, Akaike Information Criterion (AIC), Bayesian Information Criterion (BIC), the adjusted Bayesian Information Criterion (aBIC), and the interpretability and definition of classifications, where an entropy value ≥.80 represented adequate quality of classification, lower AIC, BIC, and aBIC values indicate better model fit, and the “turning point” of the scree plot for the aBIC could suggest an appropriate number of classes.

After the selection of optimal model and definition of classifications, Chi‐square test and analysis of variance began with the full set of demographic and perceived social support, depressive symptoms, and HIV knowledge to evaluate their associations with different characteristics of anticipated HIV stigma. Statistically significant variables (*p* ≤ .10) in the univariate analysis were further used for RMM analysis. In RMM, stepwise logistic regression with modal maximum likelihood algorithm was used for parameter estimation to obtain robust results (Vermunt, 2010). Adjusted odds ratio and the corresponding 95% confidence intervals were calculated to assess the RMM results.

Individuals assigned to the latent profile with the fewest symptoms or risks are referred to as “non‐cases” in LPA, whereas others are regarded as “cases” (Fu et al., 2022). These two groups serve as the “gold standard.” Based on the “gold standard,” the ROC analysis was conducted to determine the optimal cut‐off value for the AHSQ. The area under the ROC curve (AUC), sensitivity, specificity, and Youden's index value were employed to evaluate the performance of classifiers, and Youden's index value was used to identify the optimal cut‐off value (Banyai et al., 2017; Kiraly et al., 2017). SAS9.4 and Mplus8.3 were utilized to conduct all the analyses with level of significance determined at a.05 *p*‐value.

## 3 RESULTS

### 3.1 Demographic characteristics

As illustrated in Table 1, over half of the participants were less than or equal to 30 years old (*n* = 864, 61.98%) and had full‐time job (*n* = 911, 65.4%). The majority of the participants were unmarried (*n* = 1210, 86.8%) and from Han ethnicity (*n* = 1242, 89.1%). Nearly a half of the participants had bachelor or above educational level (*n* = 716, 51.4%). Most participants had an income less than 7000 yuan per month (*n* = 1013, 72.7%) and had ever has HIV test (*n* = 1029, 73.8%). More than a half of participants had sex with male in the last 6 months (*n* = 967, 69.4%), whereas only few had sex with female in the last 6 months (*n* = 159, 11.4%). Nearly a third of participants had substance use in the last 6 months (*n* = 461, 33.1%). Only 3.1% (43/1394) had history of using PrEP. Overall, 47.70% (665/1394) of the participants had depressive symptoms. The descriptions of the seven items of AHSQ are presented in Table 2.

**TABLE 1:** Demographic characteristics of the participants.

Variable	*N* (%)
**Age (Year)**	
≤30	864(61.98%)
>30	530(38.02%)
**Marriage**	
Unmarried	1210(86.8%)
Married	184(13.2%)
**Work status**	
No job or part‐time job	483(34.6%)
Full‐time job	911(65.4%)
**Ethnicity**	
Han	1242(89.1%)
Other	152(10.9%)
**Education level**	
Below bachelor	678(48.6%)
Bachelor or above	716(51.4%)
**Income (yuan/month)**	
<7000	1013(72.7%)
≥7000	381(27.3%)
**Ever had HIV test**	
No	365(26.2%)
Yes	1029(73.8%)
**Had sex with male in the last 6 months**	
No	427(30.6%)
Yes	967(69.4%)
**Had sex with female in the last 6 months**	
No	1235(88.6%)
Yes	159(11.4%)
**Substance use in the last 6 months**	
No	933(66.9%)
Yes	461(33.1%)
**History of using PrEP** No Yes	1351(96.9%) 43(3.1%)
**Depressive symptoms**	
No	729(52.30%)
Yes	665(47.70%)

Abbreviation: PrEP, pre‐exposure prophylaxis.

**TABLE 2:** Descriptive statistics of the 7 items of the Anticipated Hiv Stigma Questionnaire.

Item	Mean (SD)
If I had HIV, I'd worry about people discriminating against me	3.24 (0.86)
If I got infected with HIV, no one would date or become involved with me	3.06 (0.87)
If I got infected with HIV, no one would want to have sex with me	3.22 (0.85)
If I got infected with HIV, I would work hard to keep my HIV status a secret	3.12 (0.92)
If I got infected with HIV, I would feel set apart and isolated from the rest of the world	2.90 (0.95)
If I got infected with HIV, I would feel I was not as good a person as others	2.72 (1.03)
I would not feel ashamed of getting HIV (reverse scored)	2.76 (0.10)
Overall	3.00 (0.66)

### 3.2 Latent profile analysis

**FIGURE 1:**
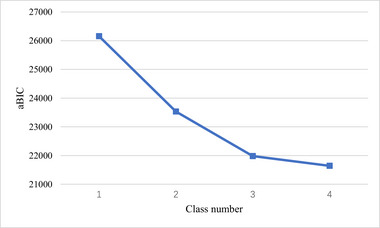
Scree plot of adjusted Bayesian Information Criterion.

**FIGURE 2:**
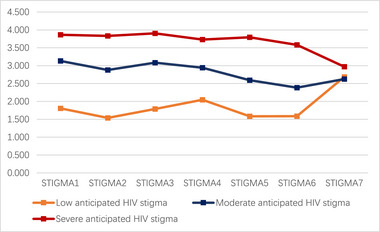
Three classes of the best‐fitting 3‐class model based on Anticipated Hiv Stigma Questionnaire.

LPA with one‐to‐four‐class solutions was specified, and the fit indices of the 4 models are displayed in Table 3. The entropies of all classifications were above 0.8. The LMR and BLRT test were statistically significant from one to three‐class model, and the LMR was not statistically significant in 4‐class model. The AIC, BIC, and aBIC decreased with the increase of class number, and the scree plot of aBIC flattened out after the 3‐class model, see Figure 1. Taken together, considering the model fit, parsimoniousness, and interpretability of the classes, the 3‐class model was selected as the optimal model for the current sample, the distribution and conditional means of items of AHSQ on each class in the 3‐class model are illustrated in Figure 2 and Table 4. In the 3‐class model, the average latent class probabilities for most likely latent class membership (0.939, 0.957, and 0.960) demonstrate reasonable classification and good distinction, see Table 5. Given the conditional means of items on each class, we define Class 1 (*n* = 167, 12.0%) as “low anticipated HIV stigma” group, class 2 (*n* = 726, 52.1%) as “moderate anticipated HIV stigma” group, and class 3 (*n* = 501, 35.9%) as “severe anticipated HIV stigma.”

**TABLE 3:** Model fit indices for latent profile models with different 1–4.

Class number	AIC	BIC	aBIC	Entropy	LMR	BLRT	Class membership probability
1	26,128.774	26,202.133	26,157.66	0			1
2	23,489.217	23,604.496	23,534.61	0.819	<0.001	<0.001	0.344/0.656
3	21,920.615	22,077.813	21,982.515	0.903	0.008	<0.001	0.120/0.521/0.359
4	21,562.742	21,761.86	21,641.148	0.899	0.062	<0.001	0.126/0.079/0.453/0.342

Abbreviations: aBIC, adjusted Bayesian Information Criterion; AIC, Akaike Information Criterion; BIC, Bayesian Information Criterion; BLRT, bootstrap likelihood ratio test; LMR, Lo‐Mendell‐Rubin.

**TABLE 4:** Conditional means of items of Anticipated Hiv Stigma Questionnaire on each class.

	Low anticipated HIV stigma	Moderate anticipated HIV stigma	Severe anticipated HIV stigma
STIGMA1	1.805	3.132	3.864
STIGMA2	1.539	2.880	3.831
STIGMA3	1.787	3.085	3.904
STIGMA4	2.047	2.943	3.729
STIGMA5	1.585	2.592	3.793
STIGMA6	1.586	2.385	3.581
STIGMA7	2.686	2.625	2.970
Class membership probability	0.120	0.521	0.359

**TABLE 5:** Average latent class probabilities for most likely latent class membership by latent class.

Latent class	Latent class membership		
	Low anticipated HIV stigma (167)	Moderate anticipated HIV stigma (726)	Severe anticipated HIV stigma (501)
Low anticipated HIV stigma	0.939	0.061	0.000
Moderate anticipated HIV stigma	0.014	0.957	0.028
Severe anticipated HIV stigma	0.000	0.040	0.960

### 3.3 Influencing factors of anticipated HIV stigma of MSM

The result of univariate analysis showed that below 30 years old (*χ* ^2^ = 5.571, *p* = .062), marriage (*χ* ^2^ = 5.729, *p* = .057), work status (*χ* ^2^ = 12.699, *p* = .002), ethnicity (*χ* ^2^ = 11.533, *p* = .003), income (*χ* ^2^ = 5.29, *p* = .071), ever having had sex with female in the last 6 months (*χ* ^2^ = 15.128, *p* = .001), depressive symptoms (*χ* ^2^ = 26.782, *p* < .001), knowledge (*F* = 5.74, *p* = .003), and social support (*F* = 7.50, *p* < .001) were associated with anticipated HIV stigma, see Table 6. These variables were further employed in RMM analysis with the “low anticipated HIV stigma” group as a reference. The results are demonstrated in Table 7. The result of RMM analysis showed that higher income (AOR = 1.626, *p* = .027) and higher levels of knowledge (AOR = 1.092, *p* < .001) were positively associated with moderate anticipated HIV stigma, whereas full‐time job (AOR = .543, *p* = .002) and social support (AOR = .958, *p* = .011) were negatively associated with moderate anticipated HIV stigma; higher income (AOR = 1.808, *p* = .009), depressive symptoms (AOR = 2.301, *p* < .001), and knowledge (AOR = 1.078, *p* = .005) were positively associated with severe anticipated HIV stigma, whereas minor ethnicity (AOR = .456, *p* = .006) and social support (AOR = .944, *p* < .001) were negatively associated with severe anticipated HIV stigma.

**TABLE 6:** Univariate analysis of influencing factors of anticipated HIV stigma of men who have sex with men (MSM).

Variable	Classification of anticipated stigma			*χ* ^2^/*F*	*p*
	Low anticipated HIV stigma	Moderate anticipated HIV stigma	Severe anticipated HIV stigma		
**Age (Year)**				5.571	.062
≤30	107	467	290		
>30	60	259	211		
**Marriage**					
Unmarried	143	645	422	5.729	.057
Married	24	81	79		
**Work status**					
No job or part‐time job	48	283	152	12.699	.002
Full‐time job	119	443	349		
**Ethnicity**					
Han	143	634	465	11.533	.003
Other	24	92	36		
**Education**					
Below bachelor	92	349	237	3.232	.199
Equal or above bachelor	75	377	264		
**Income (yuan/month)**					
<7000	131	533	349	5.29	.071
≥7000	36	193	152		
**Had sex with male in the last 6 months**					
No	46	231	150	1.343	.511
Yes	121	495	351		
**Had sex with female in the last 6 months**					
No	147	665	423	15.128	.001
Yes	20	61	78		
**Substance use in the last 6 months**					
No	112	479	342	.701	.704
Yes	55	247	159		
**Ever had HIV test**					
No	45	195	125	.616	.735
Yes	122	531	376		
**History of using PrEP** No Yes	160 7	705 21	486 15	.788	.674
**Depressive symptoms**					
No	109	399	221	26.782	<.001
Yes	58	327	280		
**Knowledge**	12.916 ± 3.912	13.847 ± 2.983	13.633 ± 3.266	5.74	.003
**Social support**	11.892 ± 6.096	10.899 ± 5.089	10.098 ± 5.741	7.50	<.001

Abbreviation: PrEP, pre‐exposure prophylaxis.

**TABLE 7:** Regression mixture model (RMM) analysis of influencing factors of anticipated HIV stigma of men who have sex with men (MSM).

Variable	Moderate anticipated HIV stigma				Severe anticipated HIV stigma			
	AOR	95% CI		*p*	AOR	95% CI		*p*
		LL	UL			LL	UL	
**Work status**								
No job or part‐time job	1				1			
Full‐time job	.543	.366	.804	.002	.745	.491	1.131	.167
**Ethnicity**								
Han	1				1			
Other	.834	.51	1.366	.471	.456	.26	.8	.006
**Income (yuan/month)**								
<7000	1				1			
≥7000	1.626	1.057	2.501	.027	1.808	1.157	2.825	.009
**Had sex with female in the last 6 months**								
No	1				1			
Yes	.873	.5	1.524	.633	1.687	.969	2.938	.065
**Depressive symptoms**								
No	1				1			
Yes	1.396	.971	2.008	.072	2.301	1.575	3.36	<.001
**Knowledge**	1.092	1.039	1.148	<.001	1.078	1.023	1.136	.005
**Social support**	.958	.928	.99	.011	.944	.912	.977	<.001

Abbreviations: AOR, adjusted odds ratio; CI, confidence intervals.

### 3.4 Receiver operating characteristic analysis

**FIGURE 3:**
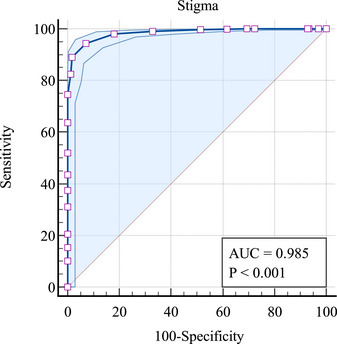
The receiver operating characteristic (ROC) curve of the Anticipated Hiv Stigma Questionnaire (AHSQ) for screening anticipated HIV stigma.

To identify the optimal cut‐off value of AHSQ for screening anticipated HIV stigma among MSM, participants assigned to the “low anticipated HIV stigma” group in LPA were defined as “non‐cases” (i.e., no stigma), and those assigned in “moderate anticipated HIV stigma” and “severe anticipated HIV stigma” groups were defined as “cases” (i.e., probable stigma). The ROC curve was then plotted for the total score of AHSQ using the binary outcome, with an AUC value of 98.50% (*p* < .001), indicating a good predictive capacity for anticipated HIV stigma, see Figure 3. The diagnostic criteria and indices are illustrated in Table 8. The optimal cut‐off value was ≥16, where the sensitivity, specificity, and Youden's index value were.945,.928, and.873, respectively.

**TABLE 8:** Criterion values and coordinates of receiver operating characteristic (ROC) curve for anticipated HIV stigma.

Criterion	Sensitivity	Specificity	Youden index
≥7	1.000	.000	.000
>7	1.000	.030	.030
>8	1.000	.066	.066
>9	1.000	.072	.072
>10	1.000	.275	.275
>11	1.000	.305	.305
>12	.999	.383	.382
>13	.998	.485	.483
>14	.990	.671	.661
>15	.980	.820	.801
**>16**	**.945**	**.928**	**.873**
>17	.890	.982	.872
>18	.824	.988	.812
>19	.746	1.000	.746
>20	.637	1.000	.637
>21	.520	1.000	.520
>22	.435	1.000	.435
>23	.376	1.000	.376
>24	.311	1.000	.311
>25	.205	1.000	.205
>26	.153	1.000	.153
>27	.102	1.000	.102
>28	.000	1.000	.000

## 4 DISCUSSION

This cross‐sectional study used LPA to identify the profiles of anticipated HIV stigma among HIV‐negative/unknown MSM in China and explored its psychosocial influencing factors with RMM. Anticipated HIV stigma was divided into 3 classes in the current study. Generally, higher income, depressive symptoms, and higher knowledge level were positively associated with anticipated HIV stigma, while having a full‐time job, minor ethnicity, and perceived social support were negatively associated with anticipated HIV stigma. The cut‐off point of AHSQ was determined at 16.

Gay identities and communities in contemporary China present a substantial contrast to numerous deep‐seated cultural values and norms. Consequently, MSM are subjected to a spectrum of stigma, predicated on their sexual orientation. This stigma can precipitate a decline in their physical and mental health. Moreover, the societal prejudice against MSM in China could have adverse implications for public health, as the apprehension of being identified or inciting suspicion may dissuade these individuals from implementing essential health precautions (Zhang et al., 2022). This study classified HIV‐negative/unknown MSM into 3 profiles based on the stigma level: “low anticipated HIV stigma,” “moderate anticipated HIV stigma,” and “severe anticipated HIV stigma.” Only 12.0% of HIV‐negative/unknown MSM were classified into “low anticipated HIV stigma” group, indicating the lowest level of stigma. The majority belonged to the “moderate anticipated HIV stigma” group (52.1%). These findings are similar to other studies of MSM that have shown high burden of stigma (Okonkwo et al., 2022). Anticipated HIV stigma of HIV‐negative/unknown MSM can negatively affect their mental and physical health and increase their experience of isolation and public embarrassment (Jiang et al., 2023). A survey conducted in Shanghai, China revealed that 97% of MSM had perceived some stigma at least once in their lifetime, and 23% had experienced at least one instance of discrimination (Liu & Choi, 2013). Therefore, the anticipated HIV stigma in this population deserves attention. Recent studies have identified several interventions that could potentially reduce HIV‐related stigma in healthcare settings. A systematic review indicated that information‐based, skill‐building, structural, contact‐based, and biomedical interventions were effective in alleviating anticipated HIV stigma in MSM (Feyissa et al., 2019). While in this study, we mainly focused on psychosocial determinants such as depressive symptoms and HIV knowledge as these factors have been identified as significant influencers in the complex dynamics surrounding stigma.

In this study, the prevalence of depressive symptoms in HIV‐negative/unknown MSM was 47.7%, higher than the result of a systematic review aimed at Chinese MSM in 2019 and an investigation among the general population in China (Lu et al., 2021; Wei et al., 2020). Additionally, the current study indicated that HIV‐negative/unknown MSM with depressive symptoms were more likely to develop severe anticipated HIV stigma. Previous research has also suggested that depressive symptoms were highly associated with anticipated HIV stigma among HIV‐negative MSM (Lo Hog Tian et al., 2021; Nouri et al., 2022; Zhai et al., 2023). Depressive symptoms experienced by HIV‐negative/unknown MSM could have negative impact on their physical, social, financial, and psychological consequence, which could deepen the fear of the possibility in HIV infection in the future (Mulqueeny et al., 2021). In addition, untreated depressive symptoms in HIV‐negative/unknown MSM could contribute to risky sexual behaviors and substance abuse, which can improve the risk of HIV infection, thus leading to severe anticipated stigma toward HIV (Turpin et al., 2021; Velloza et al., 2020; Vijayakumar et al., 2022). To alleviate depressive symptoms and anticipated HIV stigma in HIV‐negative/unknown MSM, relative department could consider implementing psychological interventions to reduce the depressive symptoms as well as anticipated HIV stigma among this population. Given the “anticipated” property of the stigma among HIV‐negative/unknown MSM, the psychological interventions could focus on alleviating their pressure from the future possibility of HIV infection, without neglecting behavioral interventions on HIV infection such as appealing condoms use in their sexual activities, and developing more convenient PrEP to protect them from infection. As a result, mindfulness‐based intervention might be a selection since it is aimed at helping people focus on the present time instead of paying too much attention on the past and the future, and this approach has proved effective to alleviate mental disorders like depression and anxiety both clinically and biologically among various populations (Diez et al., 2022; Noda et al., 2023; Slomski, 2020; Warth et al., 2022; Wells et al., 2021). Some scholars and psychiatrists have proposed a protocol on implementing a mindfulness‐based online intervention named “Mindfulness Living With Challenge” (MLWC) in COVID‐19 survivors in China (Si et al., 2021). A randomized controlled trial has also demonstrated its effect on improving the psychological well‐being of undergraduate nursing students in China (Dai et al., 2022). Therefore, health educators and relative agencies could consider conducting trials among HIV‐negative/unknown MSM to explore the effect of MLWC on alleviating anticipated HIV stigma and depressive symptoms among them.

In addition to depressive symptoms, high levels of HIV knowledge and perceived social support were both negatively associated with moderate anticipated HIV stigma among HIV‐negative/unknown MSM. A high level of HIV/AIDS knowledge among HIV‐negative/unknown MSM is essential for the implementation of prevention strategies, which will reduce their perceived risks for HIV infection and thus reduce anticipated stigma (Guimaraes et al., 2019). Furthermore, HIV‐unknown MSM are among the non‐testers and infrequent HIV testers, which is an important population targeted at knowledge improvement (Carey et al., 2021). Therefore, it is critical to improve the HIV knowledge among HIV‐negative/unknown MSM. Receiving health‐promotion materials, like online education‐entertainment intervention, was one of the most beneficial sources to acquire HIV‐related knowledge (Del Rio‐Gonzalez et al., 2021; Guimaraes et al., 2019). Perceived social support was also negatively associated with moderate HIV stigma. This result echoes previous studies that perceived social support would buffer the association between HIV symptoms and anticipated stigma among PLWH (Earnshaw et al., 2015). Higher perceived social support would help PLWH regulate their emotions and problem‐solving ability, thus focusing on the status quo and decreasing anticipated stigma (Earnshaw et al., 2013). Perceived social support could be improved from the perspective of MSM themselves and their surroundings. In terms of MSM themselves, intervention programs to enhance social skills and cognitive reframing regarding the self and social relations were found effective to improve their self‐esteem and self‐reinforcement, thus making a difference to their perception of social support (Brand et al., 1995). As for their surroundings, the support from MSM's family and friends might have an impact on MSM's perceived social support (Pan et al., 2022). With deeply ingrained traditional beliefs, some Chinese parents cannot comprehend the homosexual orientation (Ying et al., 2022). By increasing parents’ understanding and acceptance of homosexuality, MSM might have the courage to seek help from their parents and have the perception of being supported. MSM could also gain support from friends by avoiding negative relationships and making friends with people with relative knowledge.

Notably, some covariates like having no full‐time job were both positively associated with anticipated HIV stigma among HIV‐negative/unknown MSM, which were consistent with the results of previous empirical studies (Foster, 2021; Krug et al., 2019; Potts & Henderson, 2020; Suomi et al., 2022). Employment is beneficial for improving individuals’ quality of life and well‐being, which could further reduce their perceived or anticipated stigma (Lightner et al., 2021). This finding provides reference for accurate intervention among targeted population with higher risk of developing anticipated HIV stigma.

Equally important, the results of this study suggested 16 as the optimal cut‐off score for AHSQ to screen anticipated HIV stigma among HIV‐negative/unknown MSM, which provides a practical, quantifiable standard for healthcare professionals and researchers to identify and assess the anticipated level of stigma effectively among MSM and could provide guidance for further epidemiological studies on anticipated HIV stigma. Communities, relative agencies, as well as social media, are suggested to collect information on anticipated HIV stigma of HIV‐negative/unknown MSM and carry out relevant psychological intervention for those scoring exceed 16. However, currently, this scale is predominantly utilized in public health research to evaluate anticipated HIV stigma among key populations as well as in assessing the effectiveness of stigma reduction interventions. Therefore, to employ this scale in clinical settings as a tool to swiftly screen and evaluate patients’ anticipated HIV stigma is also warranted, thus enabling the implementation of tailored measures to enhance treatment outcomes.

This study enriched our understanding on the characteristics of anticipated HIV stigma and related influencing factors among HIV‐negative/unknown MSM. However, it has several limitations. First, this cross‐sectional cannot establish the causal relationship between the variables. Second, this study only recruited HIV‐negative/unknown MSM having access Blued app, which may lead to selection bias. Third, convenience sampling may affect the representativeness of our research sample. Further research could focus on the intervention on alleviating anticipated HIV stigma and improving mental health among HIV‐negative/unknown MSM in China. Fourth, the HIV‐KQ used in this study was developed in a time when prevention strategies such as PrEP were not developed. Fifth, we did not collect information such as participants’ exposure to HIV acquisition, including details about unprotected anal intercourse and HIV testing in the previous month, which might be potential indicators of anticipated HIV stigma of MSM. Further randomized controlled trials and meta analyses are also needed to validate the results of the current study. Additionally, the association between anticipated HIV stigma and the use of HIV prevention tools such as long‐acting injectable PrEP also deserves further exploration.

## 5 CONCLUSION

Anticipated stigma among HIV‐negative/unknown MSM in China could be divided into three groups: “low anticipated HIV stigma,” “moderate anticipated HIV stigma,” and “severe anticipated HIV stigma” group. Interventions on depressive symptoms, HIV knowledge, and perceived social support are suggested to alleviate anticipated HIV stigma among this population.

## AUTHOR CONTRIBUTIONS

Xiaoyou Su, Zhenwei Dai, and Yijin Wu prepared the first draft. Xiaoyou Su and Guodong Mi provided overall guidance and managed the overall project. Zhenwei Dai, Xin Liu, Jiaqi Fu, Mingyu Si, Xu Chen, Hao Wang, Weijun Xiao, Yiman Huang, and Fei Yu were responsible for the questionnaire survey and data analysis.

## CONFLICT OF INTEREST STATEMENT

The authors declare no conflicts of interest.

### PEER REVIEW

The peer review history for this article is available at https://publons.com/publon/10.1002/brb3.3472.

## Data Availability

The data that support the findings of this study are available from the corresponding author, upon reasonable request.
